# *Mytilus galloprovincialis* as a Natural Reservoir of *Vibrio harveyi*: Insights from GFP-Tagged Strain Tracking

**DOI:** 10.3390/pathogens14070687

**Published:** 2025-07-13

**Authors:** Arkaitz Almaraz, Flor O. Uriarte, María González-Rivacoba, Inés Arana, Itziar Arranz-Veiga, Beñat Zaldibar, Maite Orruño

**Affiliations:** 1 Immunology, Microbiology and Parasitology Department, Faculty of Science and Technology, University of the Basque Country (UPV/EHU), 48940 Leioa, Biscay, Spain; arkaitz.almaraz@ehu.eus (A.A.); ofrauriarte@gmail.com (F.O.U.); marivacoba@gmail.com (M.G.-R.); maite.orruno@ehu.eus (M.O.); 2 Research Centre for Experimental Marine Biology and Biotechnology (Plentzia Marine Station, PiE-UPV/EHU), 48620 Plentzia, Biscay, Spain; benat.zaldibar@ehu.eus; 3 Zoology and Animal Cell Biology Department, Faculty of Science and Technology, University of the Basque Country (UPV/EHU), 48940 Leioa, Biscay, Spain; itziar.arranz@ehu.eus

**Keywords:** *Vibrio*, mussel, reservoir, temperature, salinity

## Abstract

Vibrios are widespread in marine environments, and their persistence is often linked to natural reservoirs such as filter-feeding bivalves. This study investigated the capacity of the Mediterranean mussel, *Mytilus galloprovincialis*, to act as a reservoir of *Vibrio harveyi* using a GFP-tagged strain in controlled experiments. Mussels (shell length 4–6 cm) were exposed to *V. harveyi* gfp in estuarine and seawater at 12 °C and 20 °C over six days. Bacterial accumulation in gills, digestive gland, and gonads, as well as in feces and pseudofeces, was quantified, and the immune response following microbial challenge was assessed by histopathological analysis. Mussels actively removed *V. harveyi* from the water, but not completely. Vibrios were rapidly accumulated in organs, with the highest densities in the digestive gland (up to 10^7^–10^8^ CFU g^−1^), and substantial bacterial loads detected in biodeposits (1.55–3.77 × 10^7^ CFU g^−1^). Salinity had a greater effect than temperature on bacterial accumulation, with consistently higher counts in seawater assays. Concurrently with bacterial accumulation, mussels activated their immune system, as evidenced by the detection of granulocytomas and hemocytic infiltrations. Overall, these results demonstrate that *M. galloprovincialis* accumulates *V. harveyi* in tissues and biodeposits, serving as a natural reservoir for this bacterium.

## 1. Introduction

*Vibrio* species are ubiquitous in marine and estuarine environments, thriving in warm waters between 15 °C and 30 °C, with salinity tolerances that vary by species [1]. Their abundance fluctuates due to multiple factors, including the availability of natural reservoirs where they persist at elevated densities, such as sediments, plankton, and shellfish [2,3]. Indeed, both pathogenic and non-pathogenic *Vibrio* species have been detected in mussels and other bivalves [3,4].

*Vibrio harveyi* is a halophilic member of the Vibrionaceae family that adapts readily to environmental changes [5,6]. It is capable of infecting a wide range of fish and invertebrates worldwide [7], which establishes it as a major bacterial pathogen in marine aquaculture. This bacterium has been associated with mass mortalities in bivalves and is recognized as a pathogen to several crustacean larvae [8], notably causing luminous vibriosis in shrimp, where infected animals exhibit bioluminescence. In fish, *V. harveyi* can induce extensive lesions, including eye damage, gastroenteritis, muscle necrosis, skin ulcers, tail rot, and vasculitis [7].

The Mediterranean mussel *Mytilus galloprovincialis* is a bivalve mollusk characterized by filter-feeding, a process by which it accumulates suspended particles, including microorganisms, within its internal organs and tissues. In the filtration process, the water enters the pallial cavity through the inhalant siphon and passes through the gills, which trap the particles and direct them to the labial palps, where are sorted before entering the mouth. The discarded particles are expelled as pseudofeces, a mixture of mucus-coated particles that are ejected periodically without being digested [9]. Those particles that reach the mouth pass through the esophagus to the stomach, which is connected to the digestive gland. After digestion, the wastes are egested as feces via the exhalant siphon [10]. The fate of filtered bacteria depends on resistance to immune response and the enzymes present in the digestive system of the bivalve; thus, lysozyme-resistant bacteria are rejected without degradation [11] with feces and pseudofeces. Therefore, bivalves themselves and their biodeposits can act as reservoirs of pathogenic bacteria and may play a role in bacterial transmission in marine food chains [12].

Although bacteria are a normal component of molluscan microbiota and are essential for the health and survival of the host [13], under certain environmental conditions, they can cause pathological effects and impair the immune system [14]. Upon microbial challenge, mussels respond by triggering an inflammatory response, which involves the activation of hemocytes and granulocytes in hemolymph and some soft tissues like the digestive gland [15]. Mollusks possess only innate immunity, and the hemocytes, which share many biological functions with vertebrate macrophages, are the immune effector cells in mollusk inflammation [16]. These immune cells generate cell-mediated immunity by means of phagocytosis and the synthesis and release of lysosomal enzymes, antimicrobial peptides, and reactive oxygen species (ROS) [15,17]. The evaluation of the inflammatory response, together with quantitative histopathological analysis, can provide valuable insights into disease impacts on mussels [16,18].

The present study aims to evaluate the capacity of *Mytilus galloprovincialis*, under different conditions, to act as a reservoir of *V. harveyi* through the accumulation of this microorganism in its tissues and residual materials. In addition, the impact of *V. harveyi* on the digestive tubule profile and on the immune response of *M. galloprovincialis* was examined.

## 2. Materials and Methods

### 2.1. Vibrio harveyi Strain and Inoculum Preparation

In this study, to address the challenge of distinguishing inoculated *Vibrio* from native mussel microbiota, we used *V. harveyi* gfp, a strain of *V. harveyi* ATCC 14126^T^ modified to express the GFP (Green Fluorescent Protein) [6], enabling the specific detection and localization of the inoculated bacteria within mussel organs. The strain was stored in Microbank™ cryovials (Pro-Lab Diagnostics, Merseyde, UK) at −80 °C until use. For inoculum preparation, *V. harveyi* gfp was cultured aerobically in Marine Broth (PanReac AppliChem, Barcelona, Spain) supplemented with kanamycin (100 μg mL^−1^) at 26 °C with shaking (90 rpm) overnight to stationary phase. Cultures were harvested by centrifugation (4000× *g*, 4 °C, 15 min), washed twice with sterile saline solution (1.94% NaCl, *w*/*v*), and subsequently suspended in the same solution to a final density of approximately 2 × 10^9^ cells mL^−1^.

### 2.2. Water and Mussels

Water samples were collected from the Bay of Plentzia (43°24′54″ N 2°57’05″ W) and the Butroe river (43°22′18.06″ N, 2°54′52.01″ W), which discharges into the Plentzia estuary (Biscay Bay, northern Spain). The Plentzia estuary is a relatively shallow mesotidal system (tidal variation ~2.5 m) that constitutes the tidal section of the 7.9 km long Butroe river. It features a small leisure port near the tidal inlet, as well as the adjacent beaches of Plentzia and Gorliz. Pollution inputs are minimal; the local wastewater treatment plant (WWTP), which serves approximately 10,000 inhabitants, discharges its effluent outside the estuary through a submarine pipe extending about 1 km offshore at a depth of ~18 m [19]. The annual study carried out by the local government in terms of determining the health status of the estuary indicated that, overall, it is in good condition regarding physicochemical and biological parameters [20]. The collected samples were sequentially filtered through nitrocellulose membrane filters of 8, 0.8, 0.45, and 0.22 μm pore diameter (Millipore^®^_,_ Merck Life Science S.U.L., Madrid, Spain). To simulate estuarine conditions, artificial estuarine water was prepared by combining two parts seawater and one part river water. Both the artificial estuarine water and seawater were then sterilized by autoclaving at 121 °C for 15 min. Salinity was measured using a RES-28 ATC refractometer (Tekcoplus Ltd., Kwun Tong, Hong Kong). The resulting salinity values were 23.27 (±0.17)‰ for the artificial estuarine water and 34.9 (±0.26)‰ for the seawater. 

Prior to each experiment, approximately 70 mussels (*Mytilus galloprovincialis*) with shell lengths between 4 and 6 cm were collected at low tide in the Plentzia estuary, (43°24′33.6″ N 2°56′51.5″ W), between May and July. Individuals were gently rinsed with water from the sampling site to remove any external debris and then subjected to a depuration and acclimation process for 5 days in an open flow system before the assay. During this period, the organisms were maintained at a constant temperature of 18 °C in an aerated tank with a continuous supply of filtered natural seawater. In total, 240 mussels were used to carry out the experiments of this study.

### 2.3. Experimental Design

Artificial estuarine water and seawater were inoculated with *V. harveyi* gfp at a final density of 10^7^ cells mL^−1^ and transferred to sterile wide-mouth glass bottles (600 mL of inoculated water sample per bottle). For each experimental condition, 10 bottles were used, each containing six mussels that were randomly selected. To stimulate mussel filtration activity, 90 µL of algal suspension (*Isochrysis galbana*) at a final concentration of 10^4^ cells mL^−1^ was added. The bottles were kept without agitation but with continuous aeration throughout the trial. The experiments were conducted at 12 °C and 20 °C, representing average coastal water temperatures in the Basque Country (northern Spain) during cold and warm seasons, respectively [21]. Prior to the experiments, mussels were acclimatized for one hour at the corresponding temperature (12 °C or 20 °C), and three control mussels were collected and dissected.

Periodically (at 0, 2, 5, 10, and 20 min, and at 1, 24, and 144 h), mussels and water samples were collected. Mussels were then dissected to obtain the different organs (gills, digestive glands, and gonads) for subsequent microbiological and histological analyses (three mussels for each determination). Before and after dissection, the mussels were rinsed with sterile artificial estuarine water or seawater to remove unattached bacteria from the organs. Each organ was weighed, transferred to a tube containing 1 mL of sterile saline solution, manually crushed, and vigorously shaken prior to microbial analysis.

At the conclusion of the experiments, feces and pseudofeces were collected by filtering the surrounding water through 0.45 μm pore-diameter nitrocellulose filters (Millipore^®^). After weighing, the filters were transferred to vials containing 10 mL of sterile saline solution and vigorously shaken for 3 min.

### 2.4. Microbiological Determinations

Culturable *V. harveyi* gfp (colony-forming units, CFU g^−1^ or mL^−1^) present in organ extracts, biodeposits, or surrounding water were determined by plating on Marine Agar supplemented with kanamycin (100 μg mL^−1^). Plates were incubated in the dark at 26 °C for 24 h. Colonies emitting green fluorescence were counted under UV-A light using a UVGL-58 lamp.

The direct determination of *V. harveyi* gfp (Total Direct Count, TDC mL^−1^) in the water was carried out by epifluorescence microscopy. Formaldehyde-fixed samples (2% final concentration) were filtered through 0.22 μm pore-diameter black polycarbonate filters (Isopore^TM^_,_ Merck Life Science S.U.L., Madrid, Spain) and examined in a Nikon Eclipse E-400 epifluorescence microscope (Nikon Instruments Inc., Melville, NY, USA) equipped with a B-2A filter block (EX450-490 excitation filter, DM505 dichroic mirror, and BA520 barrier filter). Green fluorescent bacteria were counted in at least 20 randomly selected fields per sample.

### 2.5. Histological Determinations

Organs were dissected from specimens maintained in seawater at 20 °C and fixed in seawater-buffered 4% formaldehyde for 24 h. Following fixation, samples were dehydrated in an ethanol bath series, embedded in paraffin using a Leica ASP3005 tissue processor (Leica Microsystems AG, Wetzlar, Germany), and sectioned at 5 μm thickness with a Leica RM2125RTS microtome (Leica Microsystems AG) for histopathological analysis.

Some sections were stained with Hematoxylin–Eosin [22] using a Leica Autostainer XL staining station (Leica Microsystems AG) and mounted with DPX mounting medium (Merck Life Science S.U.L., Madrid, Spain) using the Leica CV5030 automatic mounter (Leica Microsystems AG). Microscopic slide observations were performed under an Olympus BX-61 light microscope (Olympus^TM^_,_ *Olympus* Iberia S.A.U.. L’Hospitalet de Llobregat, Spain). Histological sections were observed under the microscope at 40×–400× magnification and alterations were annotated. Most common lesions appeared as a diffuse hemocytic infiltration of the vesicular connective tissue or as discrete focal accumulations. In some particular cases, there was more severe inflammation (granulocytomes) within the connective tissues, replacing significant proportions of the vesicular connective tissue and digestive diverticula. The hemocytic reaction was semi-quantitatively assessed according to the scale described by Villalba et al. [23], ranging from 0 (absence of hemocytic infiltrations and granulocytomas) to 3 (presence of hemocytic infiltrations and granulocytomas).

Additionally, at least 100 digestive tubule profiles per animal were examined and classified according to their morphology: adsorbing, holding, or atrophic phase [18,24].

The remaining sections were deparaffinized, hydrated, and analyzed for the presence of fluorescent bacteria using a Nikon Eclipse Ni epifluorescence microscope (Nikon Instruments) equipped with a filter block (D395/40× excitation filter, 425DCLP dichroic mirror, and D510/40m barrier filter).

For each animal, five images of the digestive gland were captured at 200× magnification using a camera-equipped Nikon DS-Ri microscope (Nikon Instruments). All images were acquired under identical conditions of light intensity, magnification, and exposure times using the Nis Elements F software (Nikon Instruments). Subsequently, color images were converted to grayscale and segmented to determine the gray intensity of the digestive epithelia. Intensity values were converted to percentages, with 100 representing absolute white and 0 representing absolute black. These analyses were performed using the FIJI/ImageJ program (National Institutes of Health).

### 2.6. Data Processing

Colony-forming units (CFU g^−1^ or mL^−1^) and total direct counts (TDC mL^−1^) were transformed to their decimal logarithms. The arithmetic mean and the standard deviation were calculated from three replicates for each time. For histopathological studies, the normal and symmetrical distribution of the data was checked. Statistical differences between groups were evaluated using one-way ANOVA followed by Tukey’s test. A probability level of *p* < 0.05 was considered statistically significant.

## 3. Results

### 3.1. Accumulation and Distribution of V. harveyi in Mussel Organs

**Figure 1:**
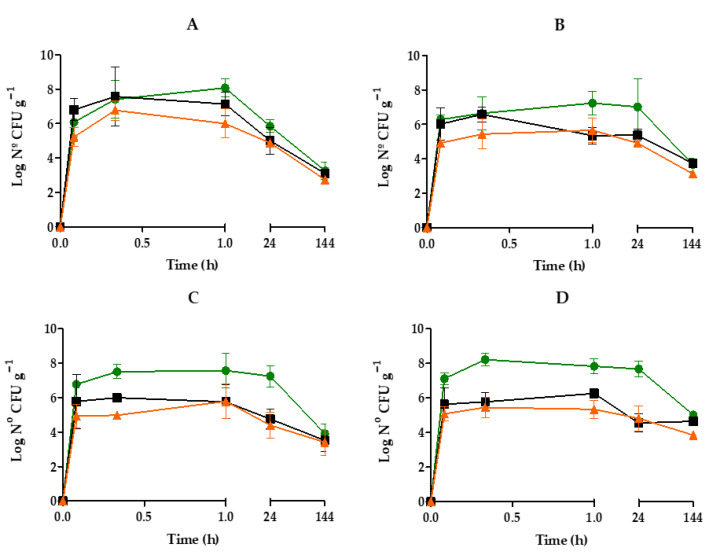
Dynamics of *V. harveyi* gfp in the different organs of *M. galloprovincialis* maintained in artificial estuarine water (**A**,**B**) and seawater (**C**,**D**) at 12 °C (**A**,**C**) or 20 °C (**B**,**D**). Gills (■), digestive gland (●), and gonads (▲). The results show the mean values of three replicates (±SD).

The number of *V. harveyi* gfp bacteria in different organs varied over time and was influenced by incubation conditions (Figure 1). Vibrios rapidly accumulated inside mussels, with high densities (>10^5^ CFU g^−1^) detected in all organs within the first 2–5 min. During the initial 20 min, differences were observed depending on water salinity. Mussels kept in seawater concentrated *Vibrio* cells preferentially in the digestive gland (10^7^–10^8^ CFU g^−1^), while those in estuarine water showed similar or higher bacterial counts in the gills compared to the digestive gland (10^6^–10^7^ CFU g^−1^). The gonads consistently showed significantly lower bacterial counts across all conditions.

Maximum *Vibrio* densities in mussels were detected between 20 and 60 min, with the digestive gland always showing the highest peak, around 10^8^ CFU g^−1^. In this organ, bacterial numbers remained high until at least 24 h, except in mussels kept in artificial estuarine water at 12 °C, where the decline started earlier, beginning after the 1st hour (Figure 1A). In the gills and gonads, bacterial counts began to decrease between 20 and 60 min. After 6 days of exposure in estuarine water, 1.2 × 10^3^, 5.5 × 10^2^, and 2.7 × 10^3^ CFU g^−1^ were enumerated in gills, gonads, and digestive glands for mussels kept at 12 °C, and 5.6 × 10^3^, 1.4 × 10^3^, and 5.8 × 10^3^ CFU g^−1^ at 20 °C. For specimens kept in seawater, these values were 5.2 × 10^3^, 4.7 × 10^3^, and 1.5 × 10^4^ CFU g^−1^ at 12 °C and 4.5 × 10^4^, 7.2 × 10^3^, and 1 × 10^5^ CFU g^−1^ at 20 °C. Overall, salinity had a greater influence than temperature on bacterial accumulation.

### 3.2. Bacterial Removal and Accumulation in Biodeposits

**Figure 2:**
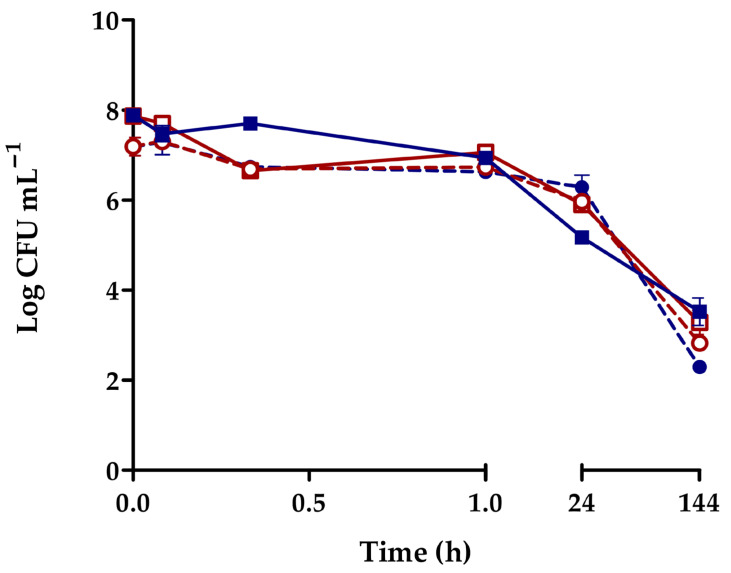
Variation of culturable *V. harveyi* gfp in surrounding water in presence of *M. galloprovincialis*. Microcosms with artificial estuarine water (■,**□**) or seawater (●,○) maintained at 12 °C (closed symbols) and 20 °C (open symbols).

Mussels actively removed *V. harveyi* from both estuarine water and seawater at 12 °C and 20 °C, as evidenced by the changes in bacterial density in the surrounding water, which remained quite stable during the first hour of exposure, gradually declining thereafter. However, a complete elimination of the vibrios was not achieved, with levels remaining above 10^2^–10^3^ cells mL^−1^ after 6 days (Figure 2). The results were consistent for both culturable and total cells. In addition, feces and pseudofeces excreted by *M. galloprovincialis* were collected and analyzed at the end of the experiments. Similar *V. harveyi* gfp densities were recovered from these biodeposits, ranging from 1.55 × 10^7^ to 3.77 × 10^7^ CFU g^−1^ across all experimental conditions.

### 3.3. Histopathology and Immune Response of Mussels Exposed to V. harveyi

**Figure 3:**
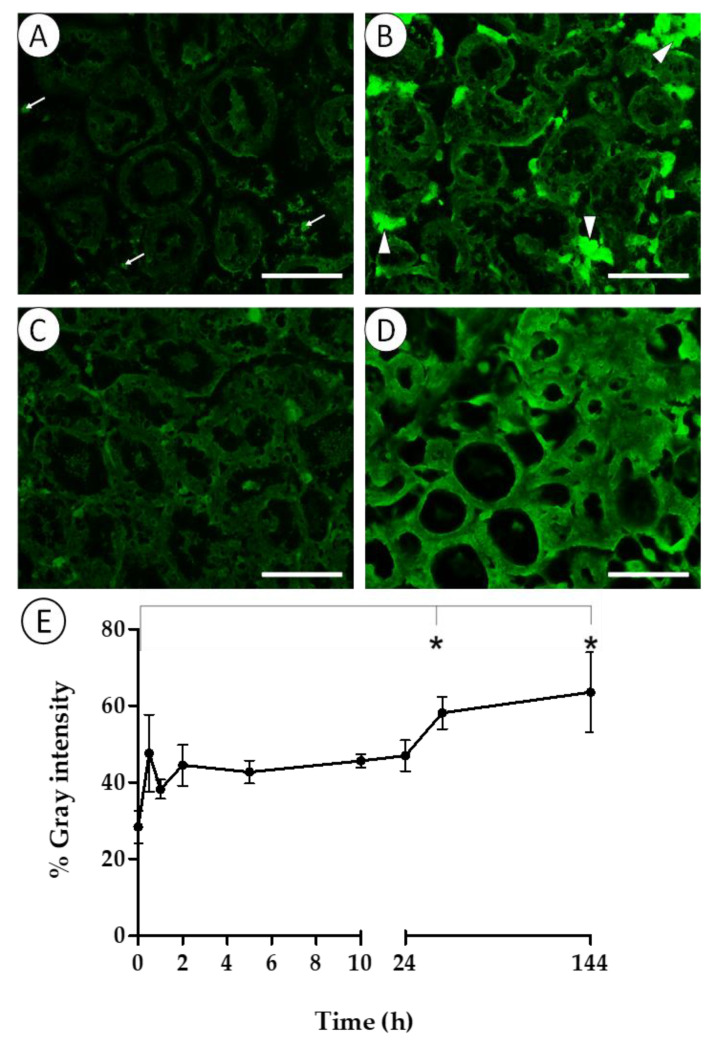
Fluorescence observed in the digestive glands of mussels maintained in seawater at 20 °C at time 0 (**A**), 2 h (**B**), 24 h (**C**), and 144 h (**D**) of exposure to *V. harveyi* gfp. White triangles indicate adipogranular cells and white arrows indicate hemocytes. Scale bar = 100 μm. Mean (±SD) fluorescence intensity converted to grayscale over the experimental time (**E**). Asterisks indicate significant differences (*p* < 0.05).

Epifluorescence microscopy revealed GFP-related fluorescence in the digestive epithelium and in cells such as hemocytes and adipogranular cells of specimens maintained in seawater at 20 °C (Figure 3A–D). The adipogranular cells showed extremely high fluorescent signals (Figure 3D); therefore, subsequent measurements focused on the epithelium of digestive diverticula. Fluorescence intensity increased after 30 min of exposure to *V. harveyi* gfp and continued to rise over time, with the highest signal intensity in the digestive epithelium after 144 h (Figure 3E). Significant differences (one-way ANOVA; F_8,15_ = 3.664; *p* = 0.013) in signal intensity were detected between 48 h and 144 h of exposure compared to the initial time point.

**Figure 4:**
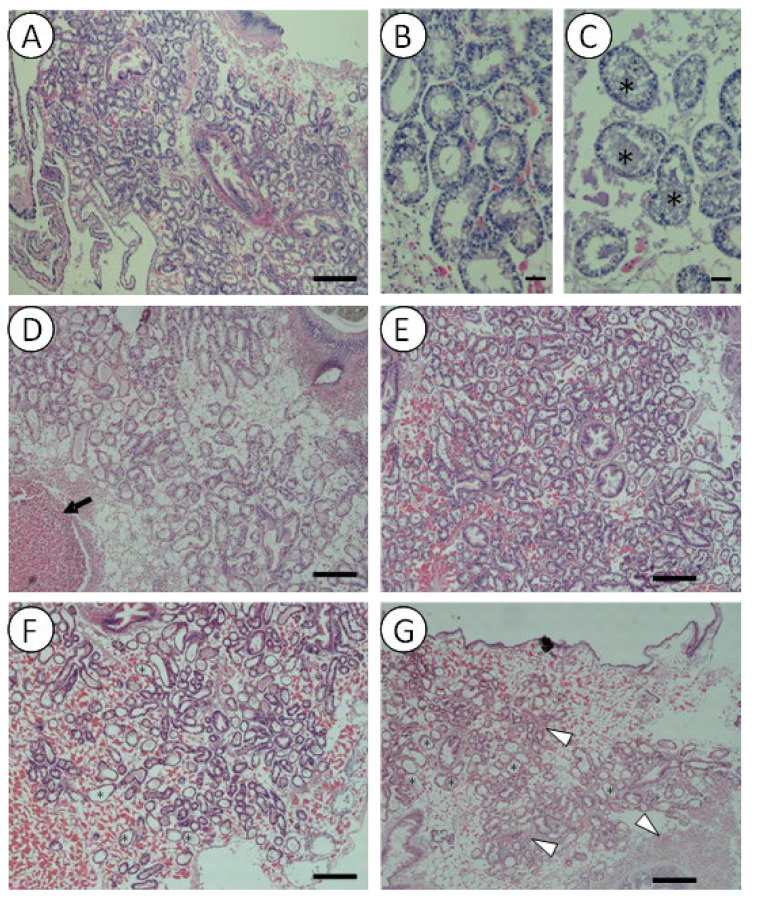
Micrographs of mussel digestive glands stained by Hematoxylin–Eosin at t0 (**A**,**B**) after 30 min (**D**), 2 h (**E**), 48 h (**C**,**F**) and 144 h (**G**) exposure to *V. harveyi* gfp in seawater at 20 °C. The white triangles indicate the hemocytic infiltrations; the black arrow indicates a granulocytoma and the asterisks, atrophic or disintegrating profiles. Scale bar = 100 μm (**A**,**D**–**G**) and 25 µm (**B**,**C**).

**Figure 5:**
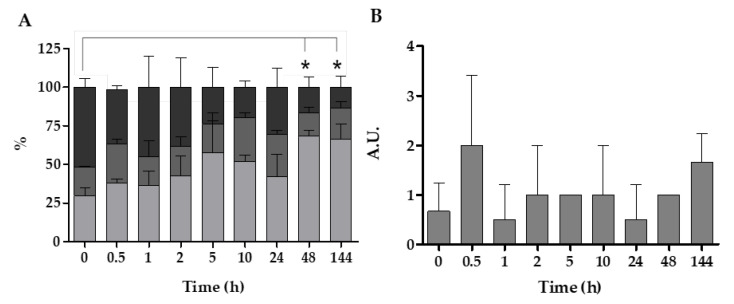
Percentage of quantification (mean ± SD) of the different phases of the digestive tubules (**A**): █ holding, █ adsorbing, █ atrophic phases. Semi-quantification (A.U., arbitrary units) of the immune response (**B**) of mussels maintained in seawater at 20 °C and exposed to *V. harveyi* gfp. Asterisks indicate significant differences (*p* < 0.05).

Histopathological analysis showed higher levels of altered health status in the digestive glands of mussels with longer exposure times to *V. harveyi* gfp. In general, after longer exposure times to *V. harveyi*, mussels presented higher interstitial connective tissue with a higher density percentage of atrophic digestive tubules or with thinner epithelium (Figure 4). Accordingly, they also presented a lower number of tubules in the resting or holding phase and an increased number of tubules in the atrophic phase, while tubules in the absorptive phase remained more or less constant throughout the experiment (Figure 5A). At this point, significant differences (one-way ANOVA; F_8,15_ = 3.802; *p* = 0.012) were also observed between 48 h and 144 h and time 0 of exposure.

Other immune system alterations, such as the presence of granulocytomas or hemocytic infiltrations, were also observed (Figure 4). The results indicate a rapid increase in the reaction of the immune system in the first 30 min of experimentation, with a slight decrease in the following hours, and a final increase after 144 h was also observed. However, the mentioned changes were not statistically significant, and immune system reactions remained mostly stable over time (Figure 5B).

## 4. Discussion

Marine bivalves are known to accumulate and concentrate different microorganisms in their tissues, and at times, they act as vectors for disease transmission [25,26,27]. Using a fluorescently labeled strain, we demonstrated in this study that the accumulation of *vibrios* is a rapid process, with the highest bacterial numbers detected in mussel organs within the first hour of exposure. This finding aligns with previous studies reporting peak accumulation times of 1 and 2 h for diverse bacterial species, including *vibrios*, in mussels [28,29] and other filter-feeding organisms such as clams [30].

Nevertheless, the existing literature provides limited insight regarding what occurs during exposure times shorter than one hour. Consequently, in the present study, we monitored the samples starting from the initial five minutes, a timeframe during which a substantial population of GFP-expressing *vibrios* was already detected within the mussel organs (Figure 1). Notwithstanding the inherent variability of mussels, the rapid appearance of vibrios in the organs is consistent with the high filtration rate of these mollusks. Indeed, assuming a filtration rate of 7.5 L h^−1^ [31], mussels are capable of filtering the entire volume of water contained in the experimental vessels in less than one minute.

The digestive gland was identified as the organ with the highest vibrio densities detected over time. This finding is not unexpected, given its critical function in food accumulation and both intracellular and extracellular digestion [32,33]. Although *vibrios* were predominantly concentrated in the digestive gland, comparable densities were also observed in the gills during the first few minutes, which reflects the importance of this organ in particle retention during filtration [9]. Similar accumulation patterns have been described in other bivalves; thus, Wang et al. [34] found the highest densities of *V. parahaemolyticus* in the digestive gland of oysters, followed by the gills.

The detection of *V. harveyi* in the gonads was lower, likely due to the contact of these organs with the pallial fluid rather than accumulation resulting from active ingestion. In natural conditions, pallial fluid contains higher bacterial densities than seawater due to its bioaccumulative capacity [35]. However, the gonads are covered by the gills and are therefore less exposed to pallial fluid.

After the initial accumulation of bacteria in mussel organs, their density began to decrease, mirroring the decline observed in the surrounding water. This reduction may result from bacterial digestion, elimination by the immune response of the mussel, or release through feces and pseudofeces. The first two processes could explain the increase in fluorescence detected in the digestive epithelium as the internal vibrio density decreased, since the lysis or digestion of vibrios would release GFP and thus increase fluorescence (Figure 3). Regardless, given the high filtration rate of mussels, the continuous presence of vibrios in the water suggests that only a fraction of the filtered microorganisms was effectively eliminated, while the remainder was returned to the medium, mainly as culturable bacteria, either free or as part of feces and pseudofeces. Accordingly, Williams et al. [36] demonstrated that part of the bacteria entering bivalves are eliminated through feces. When feces and pseudofeces are expelled, unlike the sessile organisms that generate them, they will be able to disperse via water currents, accumulate in superficial sediments, or be ingested by other organisms such as amphipods [37]. Thus, feces and pseudofeces containing pathogen microorganisms previously filtered by mussels may enter the food chain and contribute to disease dissemination [12,38].

Concurrently with bacterial accumulation in their organs, mussels responded by activating their immune system, as evidenced by the presence of alterations such as granulocytomas and hemocytic infiltrations (Figure 4 and Figure 5). This inflammatory response has been previously described in mussels exposed to different pathogens, toxins, or pollutants [18,39,40]. This process involves the recruitment of immune cells to the host tissues, which can itself cause tissue damage [41] and, consequently, may contribute to an increased percentage of atrophic digestive tubules. Furthermore, histological alterations in the digestive tubules may also result from microbial challenge, primarily affecting the digestive glands and leading to the thinning of the digestive tubule epithelium and changes in its profile and functionality [33], or even from starvation experienced by mussels during the assays.

Abiotic factors such as temperature and salinity play a role in the bioaccumulation of microorganisms in bivalves [42]. Fluctuations in salinity can influence growth, filtration rates, oxygen consumption, and immune function [43]; however, the halotolerance range remains unclear for most species [44]. In this study, salinity affected the differential retention of vibrios in the gills during the initial minutes of exposure, with lower retention observed in seawater compared to estuarine water (Figure 1). This fact may be associated with the role of *Mytilus* gills in modulating osmoregulation [45].

Previous studies have demonstrated that temperature significantly impacts the bioaccumulation of bacteria in *M. edulis* [42]. However, the literature on the effect of temperature on filtration rates has yielded conflicting conclusions. Thus, Chae et al. [46] observed higher effective depuration at slightly cold temperatures (15 °C), while Boroda et al. [47] suggested that, under cold stress, filter-feeding organisms tend to reduce their filtration rates by closing their shells as a physiological adjustment mechanism. Notably, the present study did not reveal significant differences in this regard, likely because the exposure temperatures used (12 °C and 20 °C) fall within the optimal temperature range for *M. galloprovincialis* persistence (10 °C and 32 °C) [47].

## 5. Conclusions

This study reveals that fluorescently labeled strains are a valuable tool for tracking bacterial dynamics in bivalves, enabling the precise quantification of *Vibrio harveyi* accumulation in *Mytilus galloprovincialis* organs. Our findings reveal the rapid bioaccumulation of *vibrios*, particularly in the digestive gland, in the first minutes of exposure across different environmental conditions. Although mussels responded to microbial challenge, evidenced by the presence of granulocytomas, hemocytic infiltrations, and increased fluorescence indicating bacterial digestion, *V. harveyi* persisted in tissues over time, establishing mussels as natural reservoirs. Moreover, the detection of viable *vibrios* in mussel biodeposits highlights their potential role in secondary transmission through feces and pseudofeces, emphasizing the dual ecological function of bivalves as both biofilters and vectors for aquatic pathogen dissemination.

## Data Availability

The data underlying this article will be shared on reasonable request to the corresponding author.
